# Comparative analysis of whole-genome sequencing pipelines to minimize false negative findings

**DOI:** 10.1038/s41598-019-39108-2

**Published:** 2019-03-01

**Authors:** Kyu-Baek Hwang, In-Hee Lee, Honglan Li, Dhong-Geon Won, Carles Hernandez-Ferrer, Jose Alberto Negron, Sek Won Kong

**Affiliations:** 1 School of Computer Science and Engineering, Soongsil University, Seoul, 06978 Korea; 2 Computational Health Informatics Program, Boston Children’s Hospital, Boston, MA 02115 USA; 3 Department of Pediatrics, Harvard Medical School, Boston, MA 02115 USA

## Abstract

Comprehensive and accurate detection of variants from whole-genome sequencing (WGS) is a strong prerequisite for translational genomic medicine; however, low concordance between analytic pipelines is an outstanding challenge. We processed a European and an African WGS samples with 70 analytic pipelines comprising the combination of 7 short-read aligners and 10 variant calling algorithms (VCAs), and observed remarkable differences in the number of variants called by different pipelines (max/min ratio: 1.3~3.4). The similarity between variant call sets was more closely determined by VCAs rather than by short-read aligners. Remarkably, reported minor allele frequency had a substantial effect on concordance between pipelines (concordance rate ratio: 0.11~0.92; Wald tests, *P* < 0.001), entailing more discordant results for rare and novel variants. We compared the performance of analytic pipelines and pipeline ensembles using gold-standard variant call sets and the catalog of variants from the 1000 Genomes Project. Notably, a single pipeline using BWA-MEM and GATK-HaplotypeCaller performed comparable to the pipeline ensembles for ‘callable’ regions (~97%) of the human reference genome. While a single pipeline is capable of analyzing common variants in most genomic regions, our findings demonstrated the limitations and challenges in analyzing rare or novel variants, especially for non-European genomes.

## Introduction

The clinical utility of genome sequencing has been established through the discovery of mutations in rare genetic disorders^1,2^ and treatment targets in cancer^3,4^. As such, individuals’ genome sequences are at the center of precision medicine to estimate disease risks, re-classify diseases per shared genetic risks and molecular mechanisms, and promote wellness at a population scale. To this end, coordinating efforts for large-scale genomic and clinical information such as the Global Alliance for Genomics and Health (GA4GH) is an exemplary attempt of sharing genotype and phenotype information from isolated data silos over the world^5^. One of the critical elements of sharing genotype – with or without phenotype – successfully is the accuracy and reproducibility of variant calls. However, the analytical validity of the sequencing platforms and analysis pipelines have not been fully established for whole-genome sequencing (WGS), making it difficult to compare variant calls from different sequencing platforms and pipelines.

Previous studies investigated discordant genomic variant calling results between sequencing platforms^6^, short-read aligners^7,8^, variant calling algorithms (VCAs)^9,10^, and annotation methods^11,12^. Yet, there remain outstanding issues to assure analytical validity of software pipelines^13^. First, the concordance between analytic pipelines and their performance have not been examined systematically nor comprehensively^14^. Currently, a few short-read aligners and VCAs have been developed, resulting in an even larger number of possible combinations for WGS analytic pipelines^15^. Second, previous comparison studies are limited either by narrow coverage for diverse pipelines^9,10^, by comparing only whole-exome sequencing (WES) pipelines^9,10^, or by focusing on a single European individual genome^9,16^. Finally, potential factors contributing to discordant results have not been evaluated systematically.

Here we comprehensively evaluated 70 analytic pipelines from combination of 7 short-read aligners and 10 VCAs with two WGSs from a European (HapMap sample NA12878) and an African (HapMap sample NA19240). To alleviate the bias due to relying on a single high-confidence variant call set, we used multiple high-confidence variant call sets from the Genome in a Bottle (GIAB) Consortium^14^ and the Illumina Platinum Genomes (IPG) Project^17^ for NA12878 and the catalog of variants from the 1000 Genomes Project (1KGP) Consortium^18^ for NA12878 and NA19240. Next, we investigated potential factors contributing to discordant results across pipelines using negative binomial regression. Finally, as a means to reduce false positives and false negatives in each pipeline, we tested two ensembles of pipelines combined using call concordance and unsupervised machine learning, respectively. Our results provide useful insights on variant calling using WGS to detect most variants while minimizing the risk of false negative findings.

## Results

### Concordance between analytic pipelines

For a European sample NA12878, a total of 9,120,618 variants including 8,369,894 (91.8%) biallelic variants were identified in autosomes and X chromosome from 70 pipelines. From the biallelic variants, 6,464,817 were single nucleotide polymorphisms (SNPs) and 1,670,587 were indels (Supplementary Table S1). For indels, 54 (6 short-read aligners x 9 VCAs) analytic pipelines were compared because the other pipelines did not call indels (see Methods). Similarly, in an African sample NA19240, a total of 16,293,639 variants were identified, including 15,178,990 (93.2%) biallelic ones. Among the biallelic variants, 11,802,101 were SNPs and 3,007,905 were indels (Supplementary Table S2). The number of biallelic variants identified by each pipeline was small compared to the total number of variants identified by all pipelines, confirming the variances between analytic pipelines observed in previous studies^10^. The minimum and maximum numbers of variant calls from a single pipeline is highlighted as green and orange, respectively (Supplementary Tables S1 and S2). Nonetheless, the numbers of variants identified by pipelines varied widely (max/min ratios 1.3~3.4), suggesting that the choice of pipeline could affect the sensitivity of variant calls, especially for indels (max/min ratios 2.1 for NA12878 and 3.4 for NA19240).

**Figure 1:**
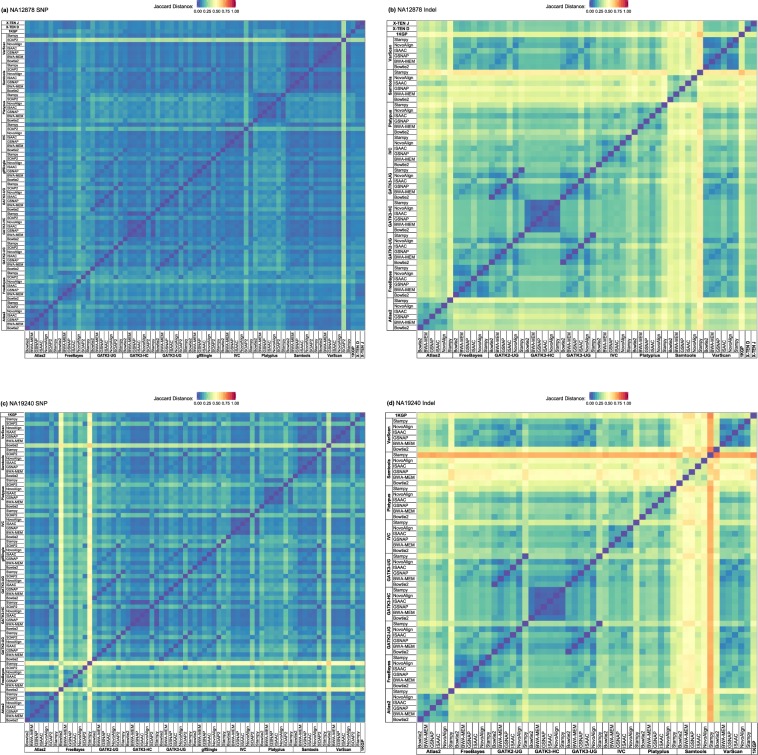
Heatmaps visualizing dissimilarity between analytic pipelines. Jaccard distances between a pair of analytic pipelines and reference variant sets from the 1000 Genomes Project (1KGP) and the Garvan Institute (X-TENs D and J) for (**a**) SNPs of NA12878, (**b**) indels of NA12878, (**c**) SNPs of NA19240, and (**d**) indels of NA19240 were respectively calculated and scaled into [0, 1].

To examine the similarity between analytic pipelines, we calculated a Jaccard distance between each pair of variant call sets from analytic pipelines (Fig. 1). For SNPs (Fig. 1a,c), we used 73 variant call sets for NA12878 (Fig. 1a; 70 analytic pipelines plus three reference variant call sets (1KGP and two variant call sets from the Garvan Institute (hereafter referred to as X-TENs; see Methods)); a total of 6,513,096 SNPs) and 71 variant call sets for NA19240 (Fig. 1c; 70 analytic pipelines plus 1KGP variant call set; contains a total of 11,832,771 SNPs). For indels (Fig. 1b,d), we used 57 variant call sets for NA12878 (Fig. 1b; based on 1,705,531 indels from 54 analytic pipelines total excluding 16 pipelines that did not call indels and three reference sets (1KGP and two X-TENs)), and 55 variant call sets for NA19240 (Fig. 1d; based on 3,027,190 indels from 54 analytic pipelines and 1KGP variant calls). Global similarities among variant call sets were largely determined by VCAs. However, the variant call sets generated using the Genome Analysis Toolkit HaplotypeCaller (GATK3-HC) clustered tightly together, presumably due to its capacity to locally re-assemble haplotypes around variants. Overall similarity among variant call sets showed greater difference by variant types (between SNPs and indels) than between individuals (NA12878 and NA19240). A similar pattern was observed from hierarchical clustering of pipelines based on the genotype call sets (see Methods; Supplementary Figs S1 and S2 for SNPs; Supplementary Figs S3 and S4 for indels).

Low depth of coverage and allelic imbalance in high-coverage NGS data are frequently associated with discordant variant calls^19^. We checked whether mean depths of coverage were different between concordant and discordant loci (see Methods). For NA12878, mean depths of coverage for concordant SNP and indel loci across all pipelines were significantly higher than those for discordant loci (Welch’s t-tests, *P* < 0.001; Supplementary Table S3 and Fig. S5). We found the same trends for NA19240 except for homozygous indels for which discordant loci had significantly higher mean depth of coverage compared to concordant homozygous indels (Welch’s t-tests, *P* < 0.001; Supplementary Table S3 and Fig. S6). For heterozygous SNPs and indels, we compared alternative allelic fractions (AFs) between concordant and discordant loci (see Methods). No significant difference was found for heterozygous SNPs in NA12878; however, concordant SNP loci had consistently higher AFs in NA19240 (Supplementary Table S3). For indels, both WGS data showed significantly higher AFs in discordant loci (Welch’s t-tests, *P* < 0.001; Supplementary Table S4). Of note, the distribution of AFs for discordant SNP and indel loci had longer tails than that for concordant loci for both NA12878 and NA19240 (Supplementary Figs S5 and S6), indicating larger variances of AFs for discordant variant loci compared to concordant ones (Supplementary Table S3).

### Sequence context and other factors contributing to concordant variant calls

**Figure 2:**
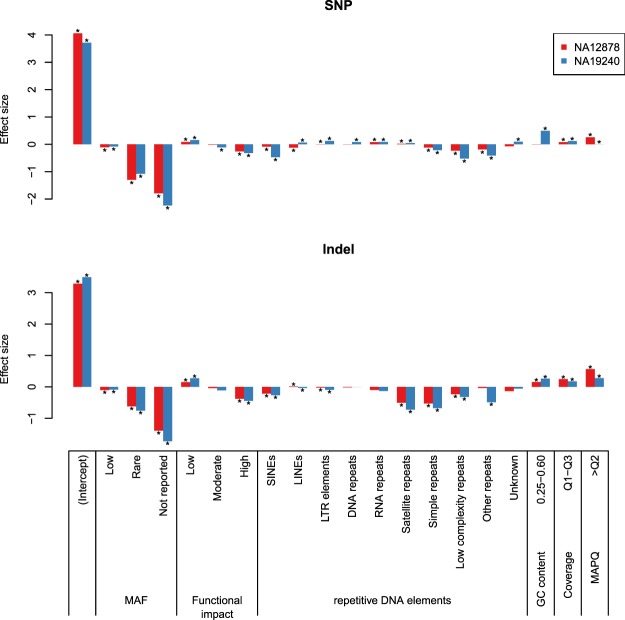
Effect size of factors related to call concordance between analytic pipelines. Negative binomial regression was performed using six factors – minor allele frequency (MAF) and predicted functional impact of variants, and repetitive DNA elements, GC content, depth of coverage, and mapping quality (MAPQ) at variant loci – to predict call concordance between analytic pipelines, i.e., the number of pipelines called a variant. Statistically significant associations (Wald tests, *P* < 0.001) are denoted by ‘*’.

The call concordance between analytic pipelines – i.e., the number of pipelines called a variant – showed a bimodal distribution as the majority of variants were called either by most pipelines or by few pipelines (Supplementary Fig. S7). On average, call concordance rates between analytic pipelines – i.e., the ratio between the number of pipelines called a variant and the number of pipelines compared – were significantly higher for NA12878 (58.1% and 34.1% for SNPs and indels, respectively) compared to those of NA19240 (40.1% and 25.0% for SNPs and indels, respectively) (Student’s t-tests, *P* < 0.001). We investigated potential factors contributing to discordant calls including minor allele frequency (MAF) and predicted functional impact of variant, as well as repetitive DNA elements, local GC content, depth of coverage, and mapping quality (MAPQ) at variant loci. To adjust for correlated factors, we performed regression analysis on relationships between the call concordance and the six factors using Poisson and negative binomial distributions, respectively (see Methods). In all cases (i.e., for SNPs and indels of NA12878 and NA19240), negative binomial regression models fitted significantly better than Poisson regression models (likelihood ratio tests, *P* < 0.001). The effect size of each factor in negative binomial regression model is shown in Fig. 2. All six factors significantly contributed to concordant variant calls between analytic pipelines (Wald tests, *P* < 0.001), with the largest effect from MAF. The variants that were not reported in the 1KGP – therefore considered as novel against the 1KGP variant call sets – showed lower concordance rates than common variants with reported MAFs in the 1KGP dataset. Notably, the concordance rate between analytic pipelines deteriorated as MAF decreased. Concordance rates for low-frequency (MAF 0.5–5%) and rare variants (MAF < 0.5%) were lower than that of common variants (MAF > 5%): x0.90–0.92 and x0.27–0.54 lower concordance rates, respectively. The concordance rate for variants with high impact as predicted by the Variant Effect Predictor (VEP)^20^ was x0.64–0.77 lower than that for variants with the least severe impact.

Among repetitive DNA elements, short interspersed nuclear elements (SINEs), simple repeats, low complexity repeats, and ‘other repeats including rolling-circles’ from the RepeatMasker database^21^ showed negative effects on concordant variant calls. In addition, indels in satellite repeats had lower concordance rates (x0.48–0.60) compared to the indels found outside of repetitive DNA elements on the human reference genome. GC content significantly influenced the call concordance, (Wald tests, *P* < 0.001) except for SNPs of NA12878. The call concordance of indels in genomic regions with normal GC content (0.25–0.60)^22^ were higher than that of the other indels (x1.17–1.30).

The depth of coverage and MAPQ of short reads significantly influenced call concordance between analytic pipelines (Wald tests, *P* < 0.001) for SNPs and indels of NA12878 and NA19240. The variants discovered in well-covered genomic regions (i.e., depth of coverage values within interquartile range for more than 80% of short-read aligners) showed × 1.08–1.28 higher concordance rates compared to the other variants. The concordance rate for variants in the genomic regions with high MAPQ scores (higher than median for > 80% of the short-read aligners) were also up to 1.76 times higher than that for variants in the other genomic region. It should be noted that sequence context – repetitive DNA elements and GC content – and the quality of short-read alignment (depth of coverage and MAPQ) independently influenced concordant variant calls, although they are presumed to be correlated with each other.

### Performance improvement by ensemble of analytic pipelines

**Figure 3:**
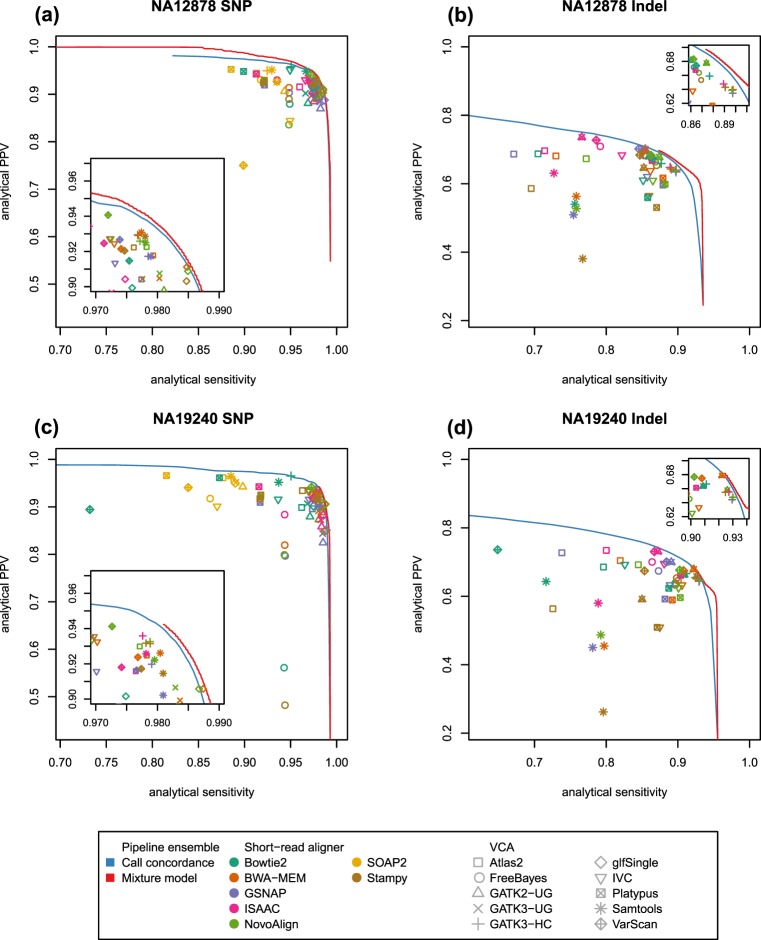
Performance comparison of analytic pipelines and their ensembles. Performances were evaluated using variant call sets from the 1000 Genomes Project for (**a**) SNPs of NA12878, (**b**) indels of NA12878, (**c**) SNPs of NA19240, and (**d**) indels of NA19240. Analytical positive predictive value (PPV) and analytical sensitivity of each pipeline without variant filtering are presented. For two ensemble methods, performance curves according to cutoff values for variant filtering are depicted. The inside plots are magnified version for clearly showing the performance of high-performance pipelines.

We first measured the performance of analytic pipelines using multiple high-confidence variant call sets and the catalog of variants from 1KGP (Supplementary Fig. S8 and Fig. 3). With variants from 1KGP as truth set, the pipelines using glfSingle as VCA achieved high analytical sensitivities and analytical positive predictive values (aPPVs) for SNPs (Fig. 3a,c), while those using GATK3-HC achieved high performance for indels (Fig. 3b,d). On the other hand, with variant call sets from GIAB and IPG as true positives, the pipelines using GATK3-HC showed high analytical sensitivities and aPPVs except for IPG SNPs (Supplementary Fig. S8). The differences in high-performance pipelines could be partly due to the differences in the genomic region containing each true variant set. The high-confidence variants in the GIAB and the IPG were respectively from 90%^14^ and 97%^17^ of the human reference genome, i.e., the ‘callable’ region for each set (see Methods). However, with the 1KGP reference call set, we used all variants from the whole region of the reference genome in the phase 3 data. As such, greater than 99% of the GIAB and the IPG variants were discovered by at least one of the 70 pipelines, while only the 94–99% of 1KGP variants were identified by one or more pipelines (Supplementary Figs S9 and S10). Moreover, the pipelines showed a wider range of performance with the 1KGP variants (Fig. 3) than with the GIAB and IPG variants (Supplementary Fig. S8).

Previous studies reported improved accuracy using an ensemble of VCAs or analytic pipelines^23–25^. We evaluated two methods for building ensembles of pipelines: call concordance-based and mixture model-based methods. The mixture model-based method used call concordance and other factors – MAF, predicted functional impact, repetitive DNA elements, GC content, depth of coverage, and MAPQ – for predicting gold standard variants (see Methods). The ensemble methods did not show any remarkable performance improvement for the GIAB and IPG variants of NA12878 except for IPG SNPs (Supplementary Fig. S8). For GIAB variants and IPG indels, the analytic pipelines using GATK3-HC showed performance comparable to or better than the ensemble methods (aPPV at the highest analytical sensitivity achievable with a single pipeline: 0.994 (‘GSNAP + GATK3-HC’) vs 0.994 (mixture model-based ensemble) for GIAB SNPs, 0.996 (‘GSNAP + GATK3-HC’) vs 0.992 (mixture model-based ensemble) for GIAB indels, and 0.991 (‘BWA-MEM + GATK3-HC’) vs 0.987 (mixture model-based ensemble) for IPG indels). The performance improvement by ensemble methods stood out for the 1KGP variants, both for NA12878 and for NA19240. For NA12878, aPPV at the highest analytical sensitivity achievable with a single pipeline was 0.888 with ‘GSNAP + glfSingle’ compared to 0.904 with the mixture model-based ensemble for SNPs, and was 0.640 (‘BWA-MEM + GATK3-HC’) vs 0.666 (mixture model-based ensemble) for indels. Similarly, aPPVs of 0.851 (‘GSNAP + glfSingle’) vs 0.896 (mixture model-based ensemble) for SNPs and 0.647 (‘BWA-MEM + GATK3-HC’) vs 0.665 (mixture model-based ensemble) for indels were obtained at the highest analytical sensitivity achievable with a single pipeline for NA19240. The mixture model-based ensemble method showed both higher analytical sensitivities and aPPVs than any single pipeline (Fig. 3).

Together with the fact that greater performance – i.e., analytical sensitivity and aPPV – variances among individual pipelines were observed for 1KGP variants than for GIAB or for IPG variants (Fig. 3 and Supplementary Fig. S8), it suggests that the factors exploited by a subset of or none of the analytic pipelines affect variant calling in the genomic regions outside the high-confidence regions by the GIAB Consortium or the IPG Project.

## Discussion

WGS is a superior genomic variant monitoring platform compared to WES due to its breadth of coverage, accuracy and a potential to identify structural variants^26–28^. However, it is challenging to process WGS to achieve analytical validity^13^. Therefore, comprehensive and continuous evaluation of next-generation sequencing (NGS) pipelines is required in the context of minimizing false negatives^29^. Previously, we have shown that detection of disease-associated variants such as rare high impact ones could be different between VCAs^25^. In the current study, we evaluated the impact of short-read alignment algorithms to final variant call sets in combination with multiple VCAs, and the performance of each analytic pipeline focusing on analytical sensitivity and aPPV. Widely used pipelines showed reasonably good performance in these terms. For instance, ‘BWA-MEM + GATK3-HC’ performed well in both analytical sensitivity and aPPV for GIAB variants of NA12878. The variability of analytic pipelines in variant calling seemed to be more influenced by VCAs than short-read aligners, suggesting that the choice of VCAs could have a substantial effect on the accuracy of variant calling. This variability is partly due to the options used for mapping and variant calling software tools. We tried to optimize the parameters of each tool; however, default parameters were used when optimization was not available.

Among the factors associated with discordant variant calls between pipelines, we observed that MAFs had a positive effect – i.e., the higher reported MAFs, the better concordance rates – on variant call concordances. Analytic pipelines produced discordant results for rare and novel variants. Similarly, lower performances were observed for rare SNPs compared to common SNPs in a study on allelic imbalance detection from quantitative sequencing data^30^. These findings suggest that reference mapping bias could be an important factor for performance degradation in sequencing-based experiments. Given the disparity in population diversity in most genomic databases (e.g., 19.5% and 6.2% in gnomAD exomes^31^ are from Asian and African origins, respectively) and higher genetic diversity in African populations due to the history of modern human migrations, we expect that the negative effect of discordant variant calls can be more pronounced in non-European individuals. Thus, pipelines with high analytical sensitivity and aPPV for a European genome could result in decreased performance when processing non-European genomes enriched with more rare SNPs and indels than a European genome. Of note, higher analytical sensitivities were observed with the pipelines with a SNP-tolerant aligner – i.e., GSNAP^32^. With increasing availability of population-scale WGS datasets, substituting SNPs with major alleles from an ancestry-matched population in the human reference genome^30^, and the refined build of the human reference genome^33^ would further improve variant calling accuracy for non-European individuals.

The current study has several limitations. First of all, we used two WGS datasets prepared with different short-read lengths (101 vs. 250 bps) and mean sequencing depths (49x vs. 72x) for NA12878 and NA19240, respectively. For NA12878, several gold standard variant call sets were used to measure the performance of each pipeline; however, we used the variant calls only from the 1KGP for NA19240. We could calculate aPPV and analytical sensitivity for the variants in a gold standard variant call set, which was not the estimation for all variants present in the genome. Therefore, our estimation of aPPV and sensitivity were upper bound. Moreover, we could not compare the performance of a single pipeline between two WGSs due to the difference in genome-wide coverage of gold standard datasets for the two individuals, as well as the difference in short-read lengths and average sequencing depths. Furthermore, variant calling accuracy fluctuates in the regions of structural variations (SVs) such as copy number variations and tandem duplications^34^; however, we did not perform SV analysis since such analysis often requires a large number of WGS sequences prepared using a same method. When comparing variant call format (VCF) files, we only left-normalized indels, and did not consider different representations of a same complex variant because our analysis focused on SNPs and indels. Finally, we did not optimize the parameters of short-read aligners and VCAs together for each of their combinations and for different short-read lengths. It is interesting to note that not all software tools are maintained to support the latest human reference genome and continuously updated to improve variant calling accuracy. For example, key features such as gVCF output and GRCh38 support were not readily available in most pipelines. gVCF output is only available for GATK3-HC and IVC (recently continued as Strelka2). Continued support and development should be considered when selecting a pipeline for a large-scale study in addition to performance at the time of testing.

We demonstrated performance differences across analytic pipelines and found that the performance from a single widely-used pipeline (e.g., using BWA-MEM followed by GATK3-HC) was not inferior to ensembles of pipelines for most genomic regions (such as high-confidence regions from GIAB or IPG) as opposed to previous reports^23–25^. Discordant results from various pipelines raised a concern regarding a choice of variant calling pipeline and potential false negatives; however, current pipelines performed reasonably well. Nevertheless, the comparative results from wider areas using 1KGP variants suggest that more sophisticated methods might be necessary for loci outside high-confidence regions, since a proportion of disease-associated variants and genes from public resources such as ClinVar and OMIM are outside the high-confidence callable region of the genome^35^.

## Methods

### WGS datasets

We downloaded WGSs of two individuals (Coriell ID: NA12878 and NA19240, respectively) from the Sequence Reads Archive (SRA) (http://www.ncbi.nlm.nih.gov/sra). The WGS dataset for NA12878 (SRA Run ID: ERR194147) was prepared using paired-end, 101-bp reads with median insert size of 300 bps (total 787,265,109 pairs) by Illumia HiSeq2000 with coverage 49x. The WGS dataset for NA19240 (SRA Run ID: ERR309934) was prepared using paired-end, 250-bp reads with median insert size of 550 bps (total 464,717,200 pairs) by Illumina HiSeq2500 with coverage 72x. The downloaded SRA files for NA12878 and NA19240 were converted into FASTQ format using the SRA Toolkit (version 2.3.5).

### Analytic pipelines

For each WGS dataset, the short reads were aligned to the human reference genome (GRCh37 with decoy sequences downloaded from the Broad Institute FTP server for the Genome Analysis Toolkit (GATK) resource bundle) using seven short-read aligners: Bowtie 2 (version 2.2.4)^36^, BWA-MEM (version 0.7.10)^37^, GSNAP (version 2014-10-22)^32^, Isaac Genome Alignment Software (ISAAC) (version 01.14.11.07)^38^, NovoAlign (version V3.02.07) (http://www.novocraft.com), SOAP2 (version 2.21)^39^, and Stampy (version 1.0.23)^40^. Stampy was used with BWA-MEM as a pre-aligner for efficient alignment as recommended by the developers of the tool. All the alignment results were stored in BAM format, except for the result from SOAP2 (which uses its own text format). The mapping result by SOAP2 was converted into BAM format using a Perl script (soap2sam.pl downloaded from http://soap.genomics.org.cn/soapaligner.html) and Samtools (version 1.1).

Each of the 14 (2 × 7) BAM files for NA12878 and NA19240 was processed by Picard tools (version 1.119) for duplicate read identification and mate-pair information verification. Then, the mapped reads were locally-realigned, and their base-quality scores were recalibrated by GATK (version 3.2-2). Finally, indels in the BAM files were left-aligned by GATK (version 3.2-2). Then, each of the BAM files was fed into 10 VCAs: Atlas2 Suite (version 1.4.3 r158)^41^, FreeBayes (version 0.9.18)^42^, GATK version 2.8-1 UnifiedGenotyper (GATK2-UG)^34^, GATK version 3.2-2 UnifiedGenotyper (GATK3-UG), GATK3-HC (version 3.2-2), glfSingle (http://csg.sph.umich.edu/abecasis/glfTools/, latest released at 2010-03-25), Isaac Variant Caller (IVC) (version 2.0.13)^38^, Platypus (version 0.7.9.1)^43^, Samtools (version 1.1)^44^, and VarScan (version 2.3.7)^45^. All the variant calling results were prepared in VCF version 4.2 (https://samtools.github.io/hts-specs/VCFv4.2.pdf). In total, we obtained 70 VCF files for each of NA12878 and NA19240. For indels, 54 pipelines were compared because the pipelines using SOAP2 or glfSingle did not support indel calling. To optimize the alignment parameters and settings for each VCA, we communicated with the original authors of each tool when possible. The exact options used for each software tool are shown in Supplementary Table S4.

### High-confidence and reference variant call sets

The GIAB VCF file for NA12878 (version 3.2.2) was downloaded from ftp://ftp-trace.ncbi.nlm.nih.gov/giab/ftp/release/NA12878_HG001/NISTv3.2.2/, which has been created by integrating multiple WES and WGS datasets, generated using four sequencing platforms^14^. The IPG VCF file for NA12878 was produced by using six analytic pipelines and two sequencing platforms for the 17 individuals of CEPH pedigree 1463^17^, and was downloaded from ftp://ussd-ftp.illumina.com/2016-1.0/hg19/small_variants/NA12878/. When evaluating performance of analytic pipelines using the GIAB and the IPG variant call sets, we focused on the respective callable regions in GRCh37 as recommended by the provider of each variant call set^14,17^. The BED files describing the callable regions were downloaded from the ftp site same as that for the VCF files. The VCF files from phase 3 of 1KGP were downloaded from ftp://ftp.1000genomes.ebi.ac.uk/vol1/ftp/release/20130502/^18^. The downloaded VCF files per chromosome were combined using VCFtools (version 0.1.12). From the combined VCF file, the 1KGP variant call sets for NA12878 and NA19240 were respectively extracted using VCFtools (version 0.1.12). Two variant call sets for NA12878 from the Garvan Institute (X-TEN sets), prepared using a latest two-color sequencing platform, Illumina HiSeq X Ten, were used as comparison call sets. These two VCF files, generated by ‘BWA-MEM + FreeBayes’ pipeline from two technical replicates of a same material, were downloaded from the repository site (http://allseq.com/knowledge-bank/1000-genome/get-your-1000-genome-test-data-set/).

### Merging multiple variant call sets and variant annotation

We merged variant call sets from analytic pipelines and the high-confidence and the reference variant call sets using BCFtools (version 1.3.1). For NA12878, 75 VCF files were merged including two high-confidence (GIAB and IPG) and three reference VCF files (1KGP and two X-TENs). For NA19240, 71 VCF files including one reference (1KGP) variant call set were merged. All variants in the two merged VCF files for NA12878 and NA19240 were annotated using the VEP release 81^20^. For efficient comparative and statistical analysis, the merged VCF files for NA12878 and NA19240 were respectively converted into the CoreArray Genomic Data Structure (GDS) using the R package *SNPRelate* (version 1.4.2)^46^. All the subsequent analysis was performed using the R statistical language^47^, bedtools (version 2.26.0), gSearch^48^, and a set of in-house C programs for matching variants in CoreArray GDS format with their VEP annotations.

### Analyzing similarity between analytic pipelines

The Jaccard distance was calculated to measure dissimilarity between each pair of variant call sets. The calculated Jaccard distance values were scaled into [0, 1] for clear visualization using heatmaps. The hierarchical clustering was used for identifying similarity structure between analytic pipelines based on genotype. Each merged VCF file (in CoreArray GDS format) was converted into a genotype matrix, in which the number of reference alleles at a locus is represented: 0 (homozygous variant), 1 (heterozygous variant), 2 (homozygous reference), and 3 (no-call). From the genotype matrix, the Euclidean distance between analytic pipelines were calculated and used for hierarchical clustering. We used R function hclust with average linkage for hierarchical clustering.

### Analyzing difference between concordant and discordant variant loci

A locus concordantly genotyped as either homozygous or heterozygous variant by all the analytic pipelines (70 for SNPs and 54 for indels) was defined as the concordant variant locus. All the other variant loci were defined as discordant. Depth of coverage for a variant locus was extracted from the field DP (i.e., read depth) for each analytic pipeline from the merged VCF files. Alternative allelic fraction was calculated using two fields – DP and AD (i.e., read depth by allele) – for each analytic pipeline excluding the pipelines using glfSingle, which did not provide any values regarding the allelic read depth.

### Poisson and negative binomial regression to identify factors contributing to call concordance between analytic pipelines

Call concordance between analytic pipelines for a variant call was defined as the number of pipelines called the variant. Based on the population-specific MAFs from 1KGP, each variant was classified as rare (MAF < 0.5%), low (0.5% ≤ MAF < 5%), common (MAF ≥ 5%), and ‘MAF not reported’. According to the severity of consequence predicted by VEP, each variant was categorized into the four groups: high, moderate, low, and modifier. As sequence context, repetitive DNA elements and GC content of a variant site were used. The RepeatMasker track from the UCSC Genome Browser^21^ was used for annotating variant sites as follows: SINEs, long interspersed nuclear elements (LINEs), long terminal repeat elements, DNA repeat elements, simple repeats, low complexity repeats, satellite repeats, RNA repeats, ‘other repeats (such as rolling-circles)’, unknown, and non-repetitive elements^49^. The GC content over a window surrounding each variant site was used for annotating the site as unbiased (25–60%) or biased GC content (>60% or <25%)^22^. The window size was set to about 2x (‘insert size’ +2x ‘read length’): 1000 bps for NA12878 and 2000 bps for NA19240. Variant sites with read-depth between the first and the third quartiles for more than 80% of the short-read aligners were annotated as having normal coverage values. The average MAPQ of the reads covering a variant site was calculated for each short-read aligner. Then, variant sites with an average MAPQ value larger than the median of the average MAPQ for more than 80% of the short-read aligners were annotated as having a good MAPQ. With six categorical predictors in total, Poisson and negative binomial regression on call concordance between analytic pipelines was performed separately for SNPs and indels, using R functions glm and glm.nb in the R package *MASS* (version 7.3–47), respectively.

### Ensemble of analytic pipelines

We combined variants called by different analytic pipelines by matching for position, reference, and alternate alleles. The matched variants were filtered either by call concordance or using a mixture model-based method. The mixture model-based method used call concordance (CC) and the six factors influencing CC, i.e., MAF, predicted functional impact (IMPACT), repetitive DNA elements (RMSK), GC content (%GC), depth of coverage (COV), and MAPQ, as variables. We used logistic regression analysis to select variables significantly associated with predicting gold standard variants (see Supplementary Methods for details). For SNPs, CC and the six factors were all significantly associated with gold standard variant prediction (see Supplementary Figs S11 and S12; Wald tests, *P* < 0.005). For indels, the variable IMPACT was not significantly associated with gold standard variant prediction for all cases (i.e., GIAB and IPG for NA12878, and 1KGP for NA12878 and NA19240) (see Supplementary Figs S11 and S12).

For SNPs, we learned a two-component mixture model using the seven variables. The two components correspond to true variant and calling error, respectively. The two-component mixture model represents the joint probability distribution over the seven variables as follows.

1 $$\begin{array}{c}p({\rm{CC}},{\rm{MAF}},{\rm{IMPACT}},{\rm{RMSK}}, \% {\rm{GC}},{\rm{COV}},{\rm{MAPQ}})\\ =\,\sum _{i=1}^{2}{\pi }_{i}\cdot {p}_{i}({\rm{CC}})\cdot {p}_{i}({\rm{MAF}})\cdot {p}_{i}({\rm{IMPACT}})\cdot {p}_{i}({\rm{RMSK}})\cdot {p}_{i}( \% \mathrm{GC})\cdot {p}_{i}({\rm{COV}})\cdot {p}_{i}({\rm{MAPQ}}),\end{array}$$

where *π*_*i*_ denotes the probability that a SNP belongs to the *i*-th component, and *p*_*i*_(·) is either a probability mass or a probability density function depending on its arguments. ${p}_{i}({\rm{CC}})$ was modeled as a Gaussian distribution. The other distributions were modeled as categorical distributions. The mixture model was learned using the expectation-maximization (EM) algorithm separately for NA12878 and NA19240. When learning mixture models for GIAB and IPG SNPs of NA12878, only the SNPs in the respective callable regions were used. The initial probability of each SNP to belong to the first component of the mixture model was set as CC/(the maximum value of CC). To prevent learning a component with extremely small variance for ${p}_{i}({\rm{CC}})$, we used a variant of the EM algorithm penalizing small variance based on a Bayesian framework^50^, setting the minimum variance of ${p}_{i}({\rm{CC}})$ as ~1. We modified R functions lcmixed (in the R package *fpc* (version 2.1–10)) and flexmix (in the R package *flexmix* (version 2.3–14)) to implement the penalized EM algorithm for the two-component mixture model for SNPs. After learning the mixture model, the component having a larger number of SNPs with the maximum CC value was set as the component for true SNPs. The posterior probability of a SNP belonging to this component was used for SNP filtering. For indels, the same mixture model but without the variable IMPACT was learned using the learning procedure same as that for SNPs.

## Supplementary information

Supplementary information

Supplementary Table S1

Supplementary Table S2

## Data Availability

The shell scripts used for alignment and variant calling including the exact options used for each software tool are available from: https://bitbucket.org/gnome_pipeline/ngspipeline. The VCF files produced by the 70 analytic pipelines are available from the supplementary website (https://gnome.tchlab.org/pipeline_comp/index.html). The other datasets generated and/or analyzed during the current study are available from the corresponding author on reasonable request.
