# Supplementary material for: Comparative analysis of whole-genome sequencing pipelines to minimize false negative findings
Source: Sci Rep. 2019 Mar 1;9:3219. doi: 10.1038/s41598-019-39108-2 (PMC6397176; doi:10.1038/s41598-019-39108-2)
Supplement: Supplementary file 1 — Supplementary information [file 41598_2019_39108_MOESM1_ESM.docx]

Comparative analysis of whole-genome sequencing pipelines to minimize false negative findings

(Supplementary information)

Kyu-Baek Hwang^1,#^, In-Hee Lee^2,#^, Honglan Li^1^, Dhong-Geon Won^1^, Carles Hernandez-Ferrer^2^, Jose Alberto Negron^2^, Sek Won Kong^2,3,*^

1. School of Computer Science and Engineering, Soongsil University, Seoul 06978, Korea

2. Computational Health Informatics Program, Boston Children’s Hospital, Boston, MA 02115, USA

3. Department of Pediatrics, Harvard Medical School, Boston, MA 02115, USA

**#** These authors contributed equally to this work.

* Correspondence and requests for materials should be addressed to S.W.K. (email: [sekwon.kong@childrens.harvard.edu](mailto:sekwon.kong@childrens.harvard.edu))

**Supplementary Table S1.** Summary of variants called by 70 analytic pipelines for NA12878. For indels, 54 pipelines were compared because the pipelines using SOAP2 or glfSingle did not support indel calling. The minimum and maximum numbers of variant calls from a single pipeline is highlighted as green and orange, respectively.

(Provided as a separate MS Excel file)

**Supplementary Table S2.** Summary of variants called by 70 analytic pipelines for NA19240. For indels, 54 pipelines were compared because the pipelines using SOAP2 or glfSingle did not support indel calling. The minimum and maximum numbers of variant calls from a single pipeline is highlighted as green and orange, respectively.

(Provided as a separate MS Excel file)

Supplementary Table S3. Read depth for SNP and indel loci, and alternative allelic fraction for heterozygous SNP and indel loci.

| Sample | Variant type | Zygosity | NA12878 | | | NA19240 | | |
| --- | --- | --- | --- | --- | --- | --- | --- | --- |
|  |  |  | Concordant^1^ | Discordant^1^ | Sig^2^ | Concordant^1^ | Discordant^1^ | Sig^2^ |
| Read depth | SNP | Homozygous | 46.9957  ± 10.2428 | 38.0061  ± 81.2252 | *** | 61.6384  ± 21.5708 | 50.9818  ± 179.2712 | *** |
|  |  | Heterozygous | 48.3416  ± 10.3542 | 45.2253  ± 96.1552 | *** | 66.3062  ± 20.9924 | 57.2359  ± 151.9346 | *** |
|  | Indel | Homozygous | 45.6514  ± 10.1718 | 37.1727  ± 20.8078 | *** | 53.2518  ± 25.0665 | 58.0408  ± 106.1644 | *** |
|  |  | Heterozygous | 48.4444  ± 9.8142 | 38.5421  ± 26.0650 | *** | 66.5694  ± 286.2542 | 61.0490  ± 114.3048 | *** |
| Alternative allelic fraction | SNP | Heterozygous | 0.4900  ± 0.0758 | 0.4900  ± 0.2342 |  | 0.5010  ± 0.0689 | 0.4764  ± 0.1716 | *** |
|  | Indel | Heterozygous | 0.4777  ± 0.0730 | 0.5866  ± 0.2614 | *** | 0.4631  ± 0.0989 | 0.4870  ± 0.1384 | *** |
| 1 Values are represented as (mean) ± (standard deviation).  2 Welch’s two-sample t-test (two-sided). *** for *P* < 0.001, ** for *P* < 0.01, * for *P* < 0.05. | | | | | | | | |

Supplementary Table S4. Detailed command-line options for analytic pipelines.

| **Short-read Aligners** | **Detailed command-line options** |
| --- | --- |
| Bowtie2 | bowtie2 -p 6 -x $REF -1 $READ1 -2 $READ2 --rg-id "$ID" --rg "SM:$ID" --rg "PL:Illumina" \| samtools view -bS - \| samtools sort -@ 2 -m 2G -o $outputBAM -T ${tempDir} |
| BWA-MEM | bwa mem -M -t 6 -R "@RG\tID:$ID\tSM:$ID\tPL:Illumina" $REF $READ1 $READ2 \| samtools view -bS - \| samtools sort -@ 2 -m 2G -o $outputBAM -T $tempDir |
| GSNAP | gsnap --gunzip -D $REF -d b37 -t 5 -A sam --npath=1 -B 5 --read-group-id $ID --read-group-name $ID --read-group-platform Illumina $READ1 $READ2 \| samtools view -bS - \| samtools sort -@ 2 -m 2G -o $outputBAM -T $tempDir |
| ISAAC | isaac-align --temp-directory $tempDir -r $REF -b $fastqDir -m 50 -j 6 --base-calls-format fastq-gz --bam-header-tag "@RG\tID:$ID\tSM:$ID\tPL:Illumina" -o $outputDir --verbosity 3 --input-parallel-load 6 --temp-parallel-load 6 |
| NovoAlign | novoalign -c 8 --mmapoff -t 20,3 --softclip 20 -d $REF -f $READ1 $READ2 -i 350 50 -k -o SAM "@RG\tID:$ID\tSM:$ID\tPL:Illumina" \| samtools view -Sb - \| samtools sort -@ 8 -m 2G -o $outputBAM -T $tempDir |
| SOAP2 | soap -p 8 -a $READ1 -b $READ2 -D $REF -o $outputPE -2 $outputSE -u $outputUnmapped;  perl soap2sam.pl -p -s $outputPE -o $outputSAM -x $outputPE;  samtools view -bS $outputSAM \| samtools sort -@ 8 -m 2G -o $outputBAM |
| Stampy | python stampy.py -g $REF -h $REF -t8 --readgroup=ID:$ID,SM:$ID,PL:Illumina --bamkeepgoodreads -M $bwaBAM \| samtools view -bS - \| samtools sort -@ 8 -m 2G -o $outputBAM -T $tempDir |
| * program versions: Bowtie2 (version 2.2.4), BWA-MEM (version 0.7.10), GSNAP (version 2014-10-22), ISAAC (version 01.14.11.07), NovoAlign (version V3.02.07), SOAP2 (version 2.21), Stampy (version 1.0.23)  † $REF: reference genome sequence or its index, $READ1/$READ2: short-read files, $ID: sample identifier, $tempDir: temporary directory, $outputBAM/$outputSAM: aligner output in BAM/SAM format, $fastqDir: directory for short-read files, $outputPE/$outputSE/$outputUnmapped: initial output from SOAP2 (paired-ended, single-ended, unmapped), $bwaBAM: alignment by BWA | |
| **Variant Calling Algorithms** | **Detailed command-line options** |
| Atlas2 Suite | (SNPs) ruby Atlas-SNP2.rb -i $inputBAM -r $REF -o $outputVCF -n $ID --Illumina  (Indels) ruby Atlas-Indel2.rb -b $inputBAM -r $REF -o $outputVCF -I -s $ID |
| FreeBayes | freebayes -f $REF $inputBAM > $outputVCF |
| GATK2-UnifiedGenotyper | java -Xmx6g -XX:ParallelGCThreads=4 -Djava.io.tmpdir=$tempDir -jar GenomeAnalysisTK.jar -R $REF -T UnifiedGenotyper -glm BOTH --num_threads 4 -I $inputBAM --dbsnp $dbsnpVCF -o $outputVCF -stand_call_conf 50.0 -stand_emit_conf 10.0 -dcov 200 |
| GATK3-UnifiedGenotyper | java -Xmx6g -XX:ParallelGCThreads=4 -Djava.io.tmpdir=$tempDir} -jar GenomeAnalysisTK.jar -R $REF -T UnifiedGenotyper -glm BOTH --num_threads 4 -I $inputBAM --dbsnp $dbsnpVCF -o $outputVCF |
| GATK3-HaplotypeCaller | java -Xmx14g -XX:ParallelGCThreads=7 -Djava.io.tmpdir=$tempDir -jar GenomeAnalysisTK.jar -R $REF -T HaplotypeCaller -I $inputBAM --dbsnp $dbsnpVCF -o $outputVCF |
| glfSingle | glfSingle -g $inputGLF -b $outputVCF |
| IVC | configureWorkflow.pl --bam $inputBAM --ref=$REF --config=$outputDir/config1.ini --output=$outputDir; cd $outputDir && make -j 4 |
| Platypus | python Platypus.py callVariants --refFile $REF --bamFiles $inputBAM --output $outputVCF |
| Samtools | samtools mpileup -ugf $REF $inputBAM \| bcftools call -vmO v -o $outputVCF |
| VarScan | samtools mpileup -f $REF $inputBAM \| java -Xmx7g -XX:ParallelGCThreads=2 -Djava.io.tmpdir=$tempDir -jar VarScan.v2.3.7.jar mpileup2cns --output-vcf 1 --variants > $outputVCF |
| * program versions: Atlas2 Suite (version 1.4.3 r158), FreeBayes (version 0.9.18), GATK2-UnifiedGenotyper (version 2.8-1), GATK3-UnifiedGenotyper (version 3.2-2), GATK3-HaplotypeCaller (version 3.2-2), glfSingle (released at 2010-03-25), IVC (version 2.0.13), Platypus (version 0.7.9.1), Samtools (version 1.1), VarScan (version 2.3.7)  † $REF: reference genome sequence, $ID: sample identifier, $tempDir: temporary directory, $inputBAM/$inputGLF: input alignment in BAM/GLF (only in glfSingle), $outputVCF: output variant calls in VCF, $dbsnpVCF: dbSNP variants in VCF (we used dbSNP v138 from GATK 2.8 resource bundle) | |


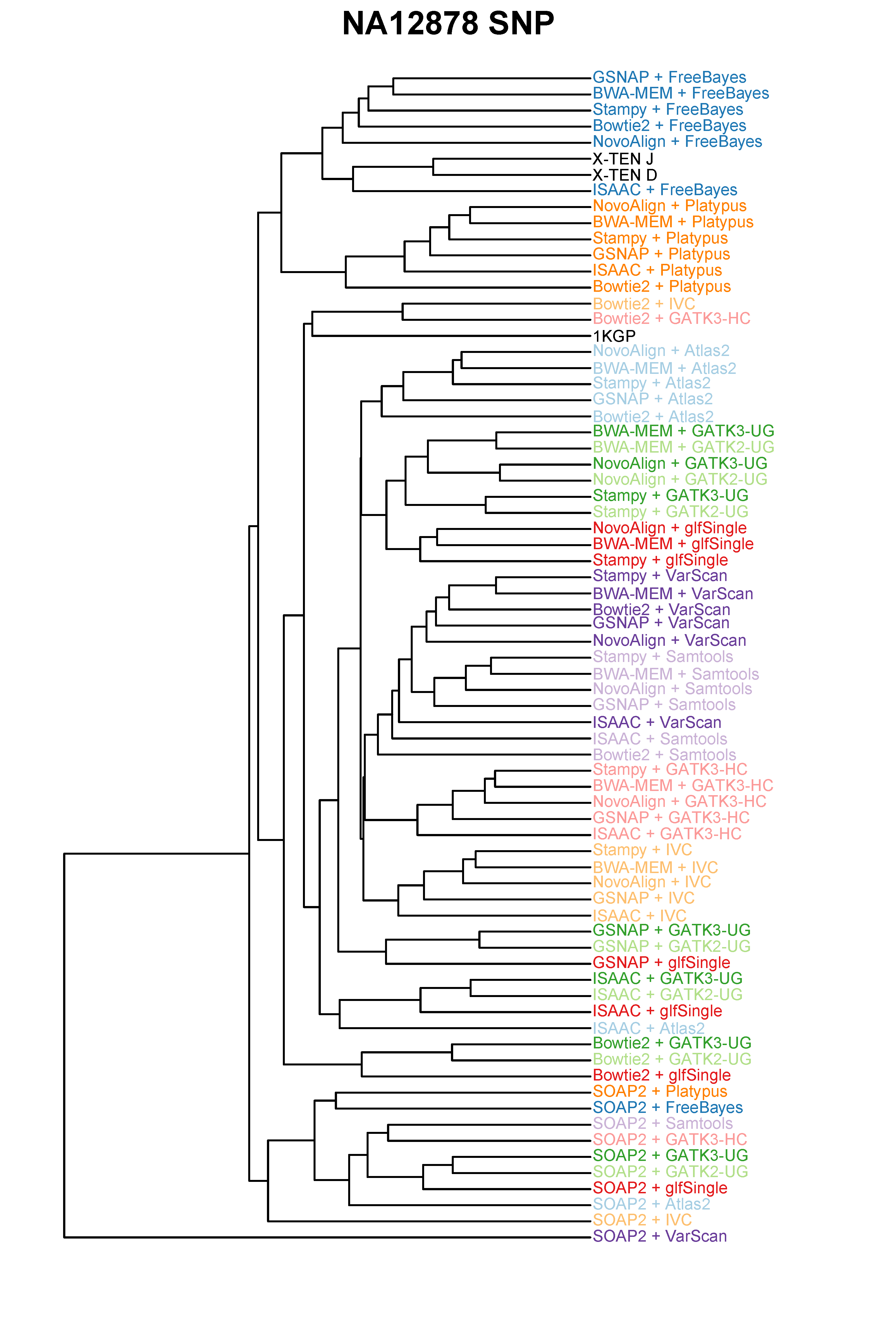


Supplementary Figure S1. Dendrogram for average linkage hierarchical clustering of 70 analytic pipelines and three reference variant sets from the 1000 Genomes Project (1KGP) and the Garvan Institute (X-TENs D and J) for SNPs of NA12878. Euclidean distances based on genotype were used for the clustering. Pipelines are shown by different colors for the variant calling algorithms used.


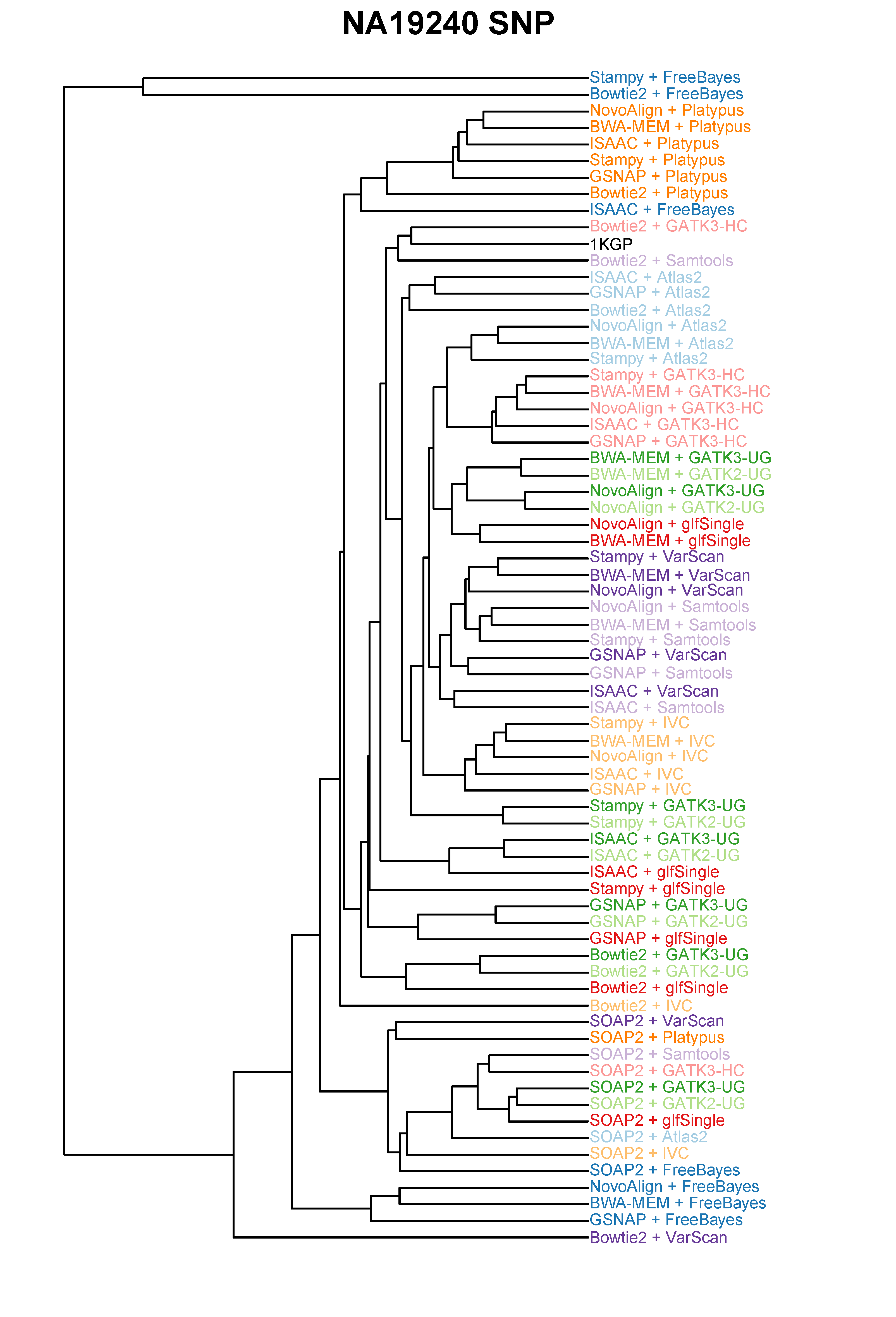


Supplementary Figure S2. Dendrogram for average linkage hierarchical clustering of 70 analytic pipelines and one reference variant set from the 1000 Genomes Project (1KGP) for SNPs of NA19240. Euclidean distances based on genotype were used for the clustering. Pipelines are shown by different colors for the variant calling algorithms used.


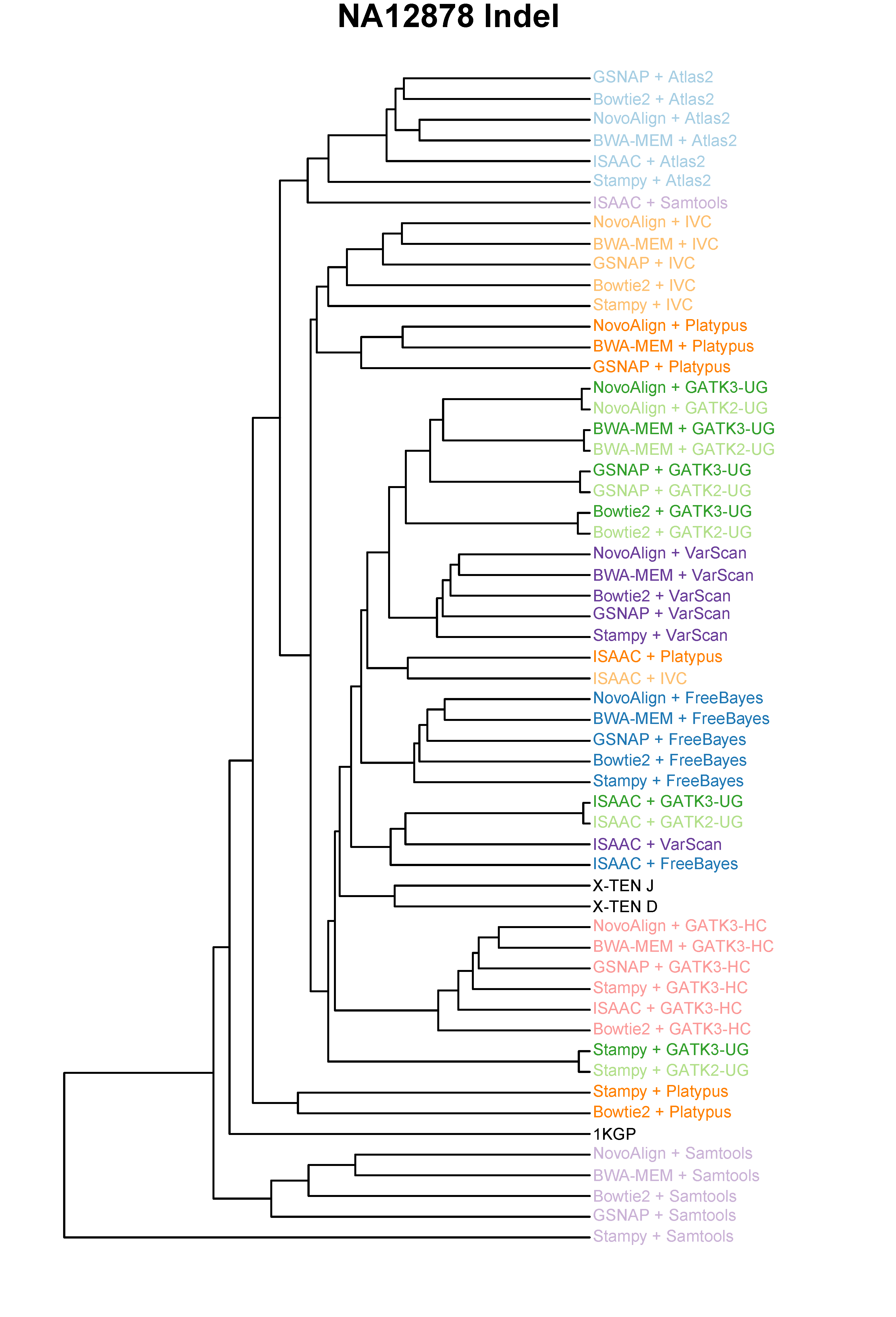


Supplementary Figure S3. Dendrogram for average linkage hierarchical clustering of 54 analytic pipelines and three reference variant sets from the 1000 Genomes Project (1KGP) and the Garvan Institute (X-TENs D and J) for indels of NA12878. Euclidean distances based on genotype were used for the clustering. Pipelines are shown by different colors for the variant calling algorithms used.


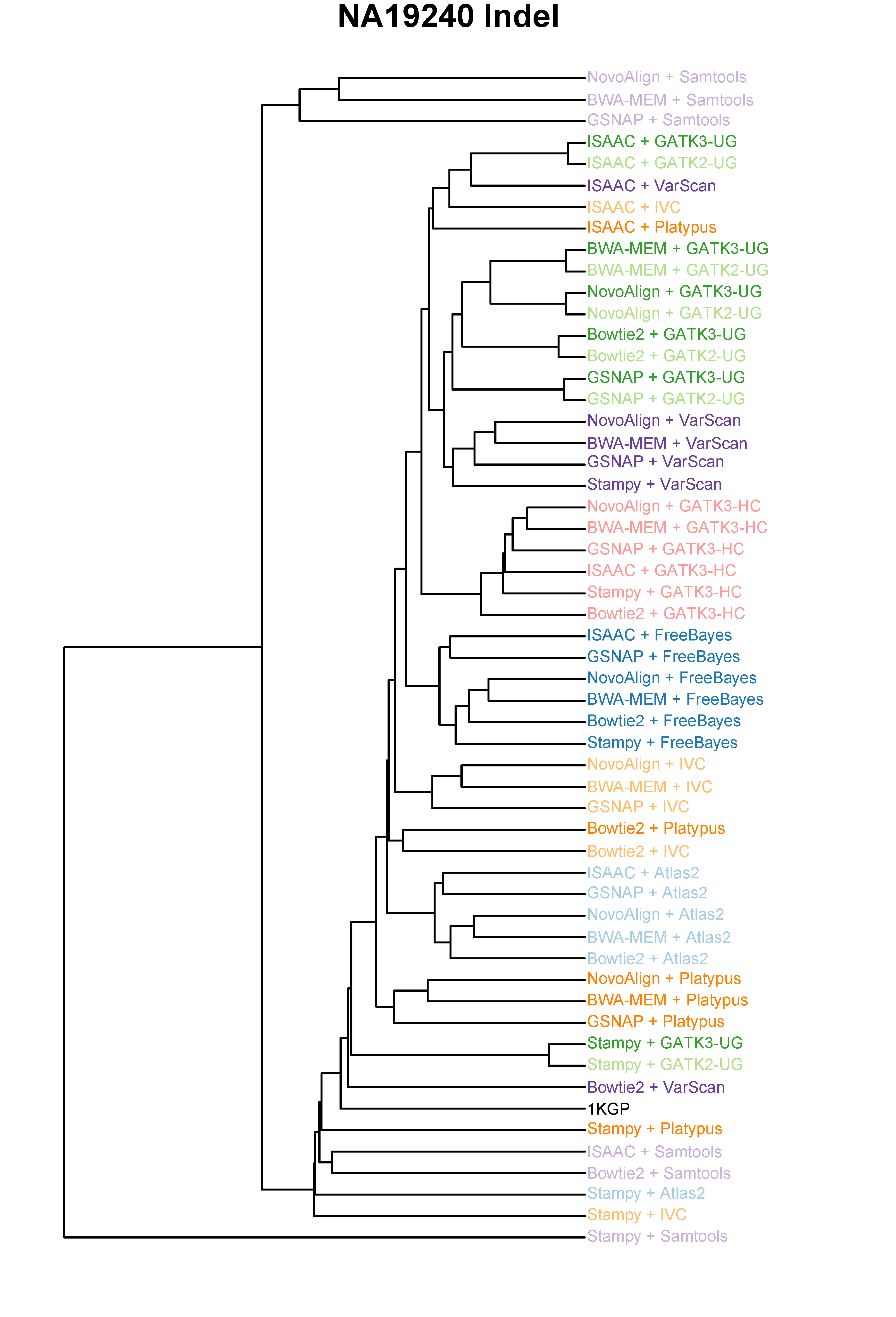


Supplementary Figure S4. Dendrogram for average linkage hierarchical clustering of 54 analytic pipelines and one reference variant set from the 1000 Genomes Project (1KGP) for indels of NA19240. Euclidean distances based on genotype were used for the clustering. Pipelines are shown by different colors for the variant calling algorithms used.

**
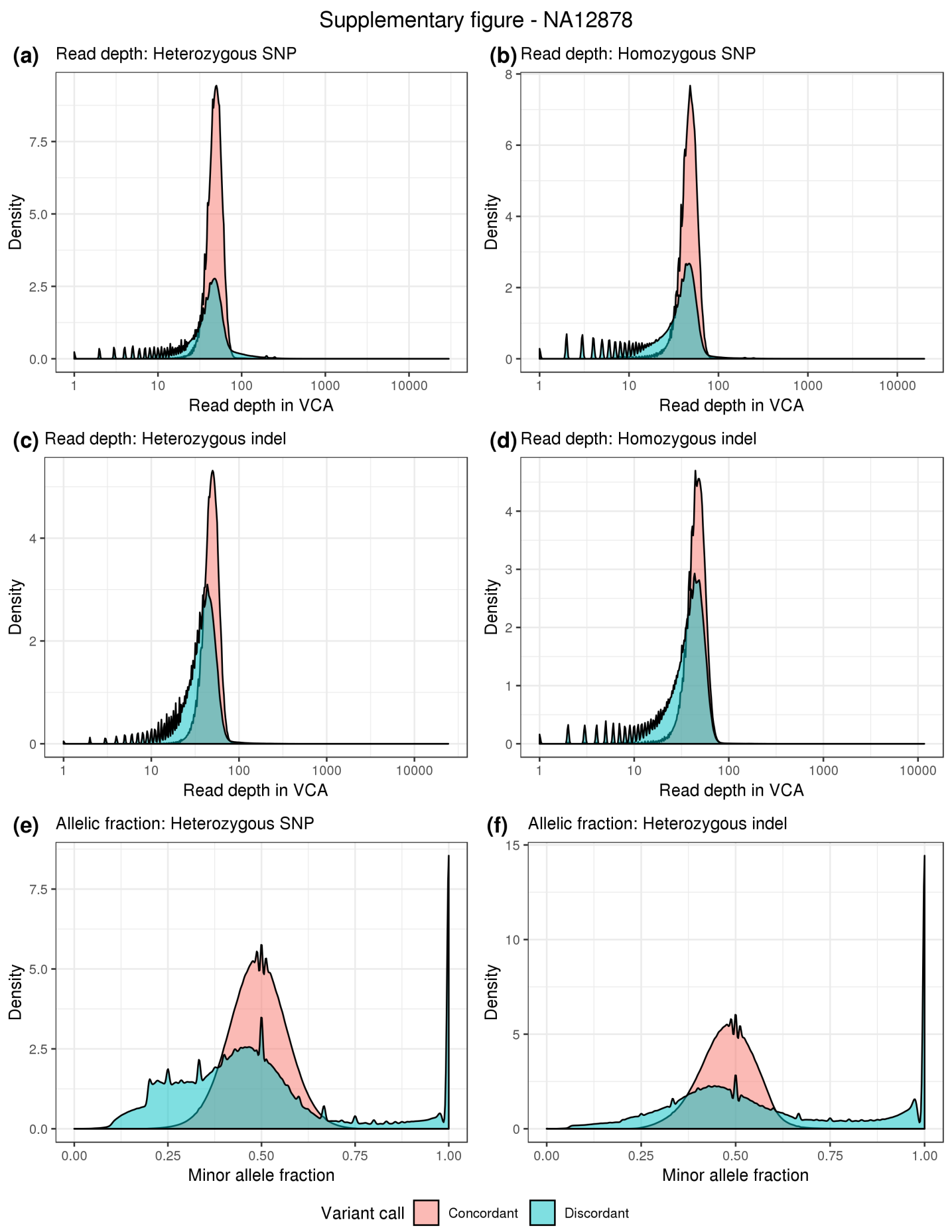
**

Supplementary Figure S5. Distribution of read depth for SNP (a and b) and indel (c and d) loci, and distribution of minor allele fraction for heterozygous SNP (e) and indel (f) loci of NA12878.

**
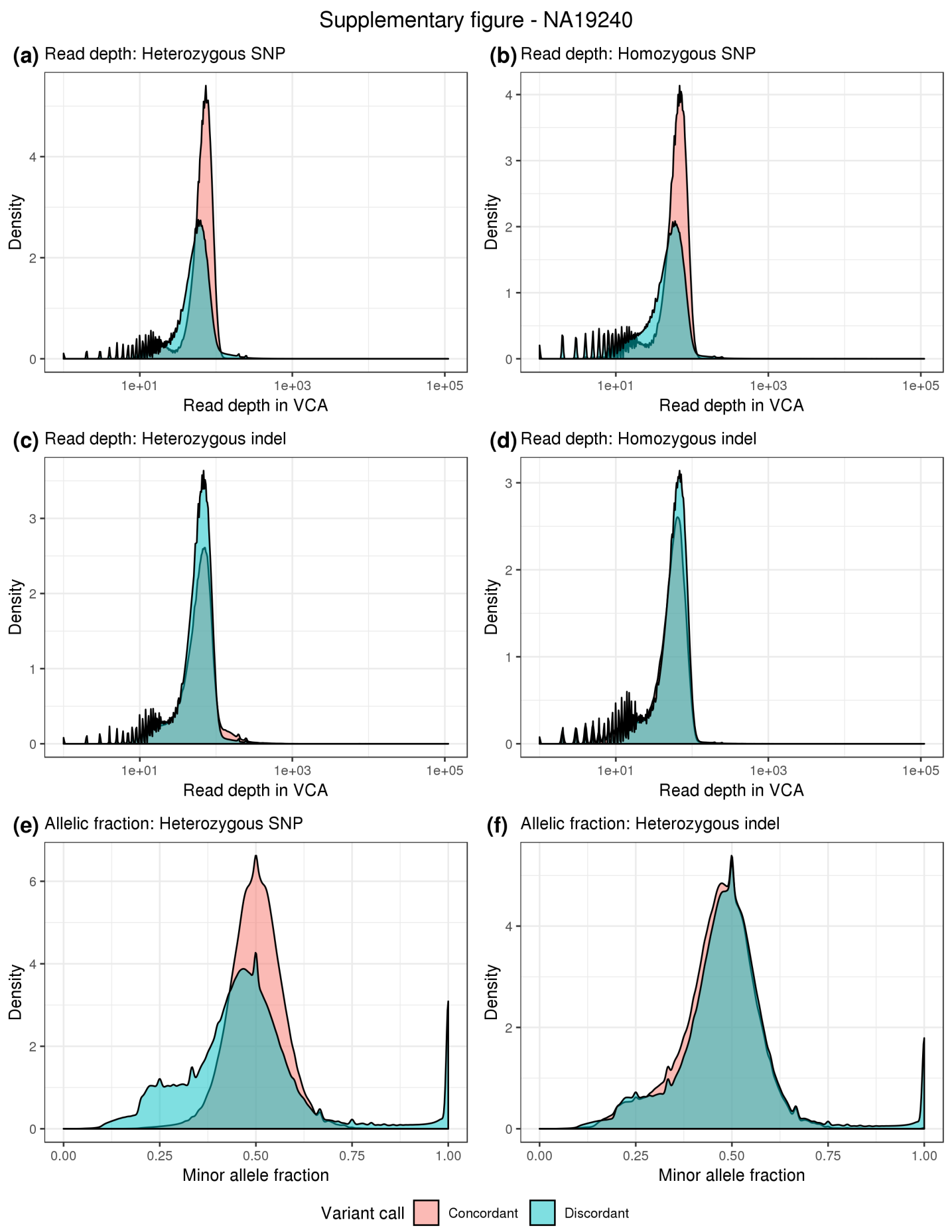
**

Supplementary Figure S6. Distribution of read depth for SNP (a and b) and indel (c and d) loci, and distribution of minor allele fraction for heterozygous SNP (e) and indel (f) loci of NA19240.


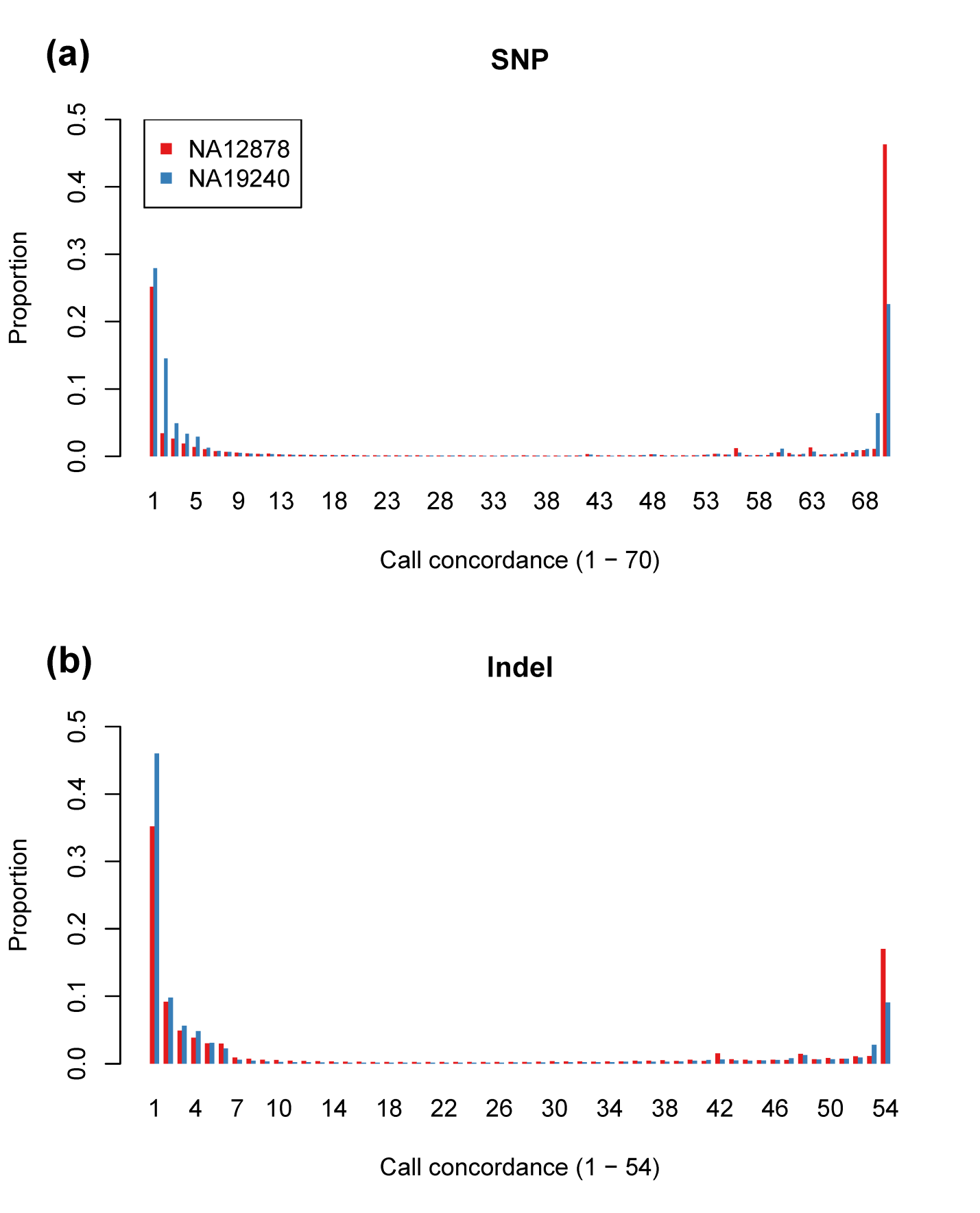


Supplementary Figure S7. Distribution of concordance on (a) SNP and (b) indel calls between analytic pipelines. 70 and 54 pipelines were compared for SNPs and indels, respectively.


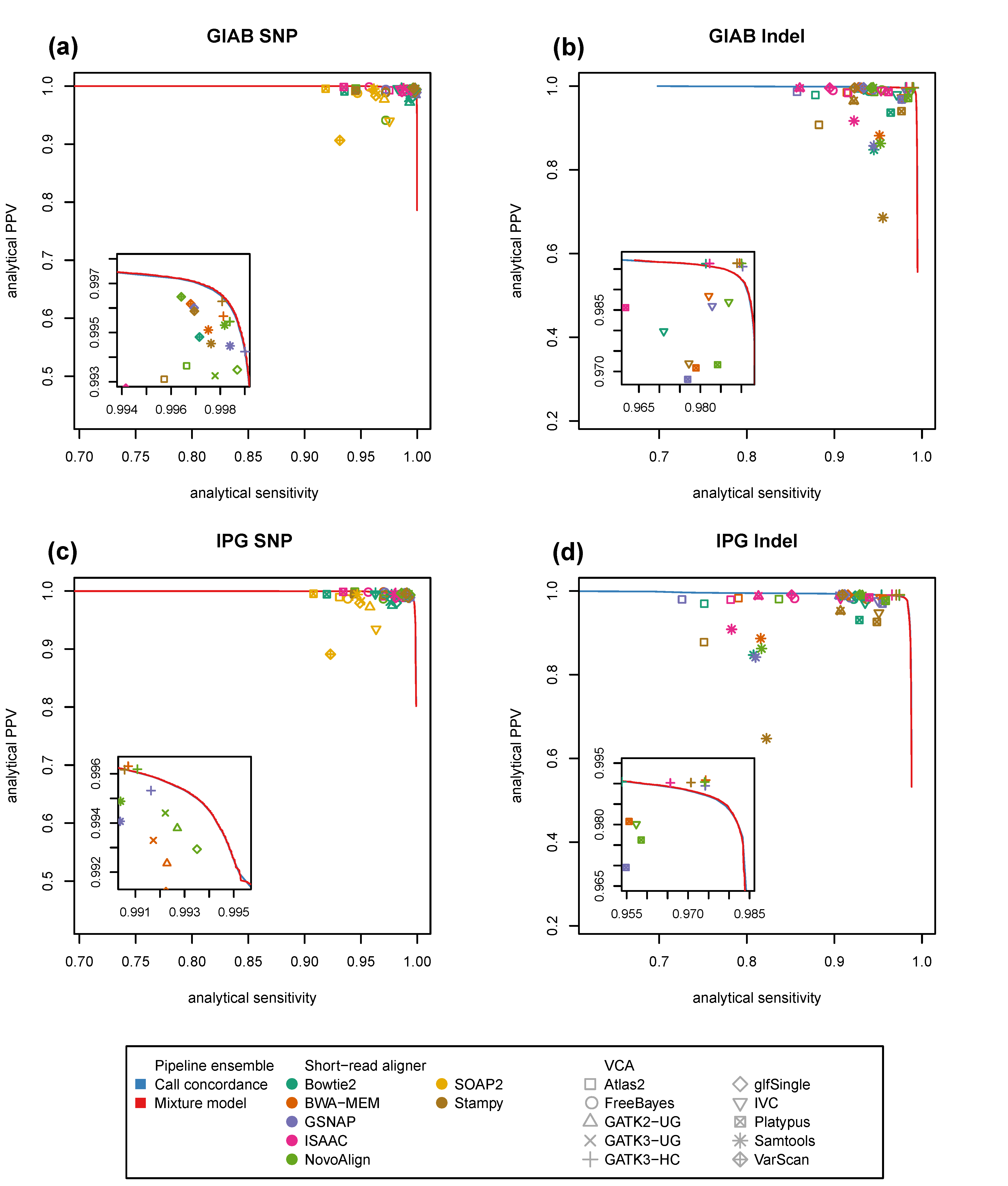


Supplementary Figure S8. Performance comparison of analytic pipelines and their ensembles for NA12878. Performances were evaluated using variant call sets from the Genome in a Bottle (GIAB) Consortium (a and b) and the Illumina Platinum Genomes (IPG) Project (c and d). Analytical positive predictive value (PPV) and analytical sensitivity of each pipeline without variant filtering are presented. For two ensemble methods, performance curves according to cutoff values for variant filtering are depicted. The inside plots are magnified version for clearly showing the performance of high-performance pipelines.


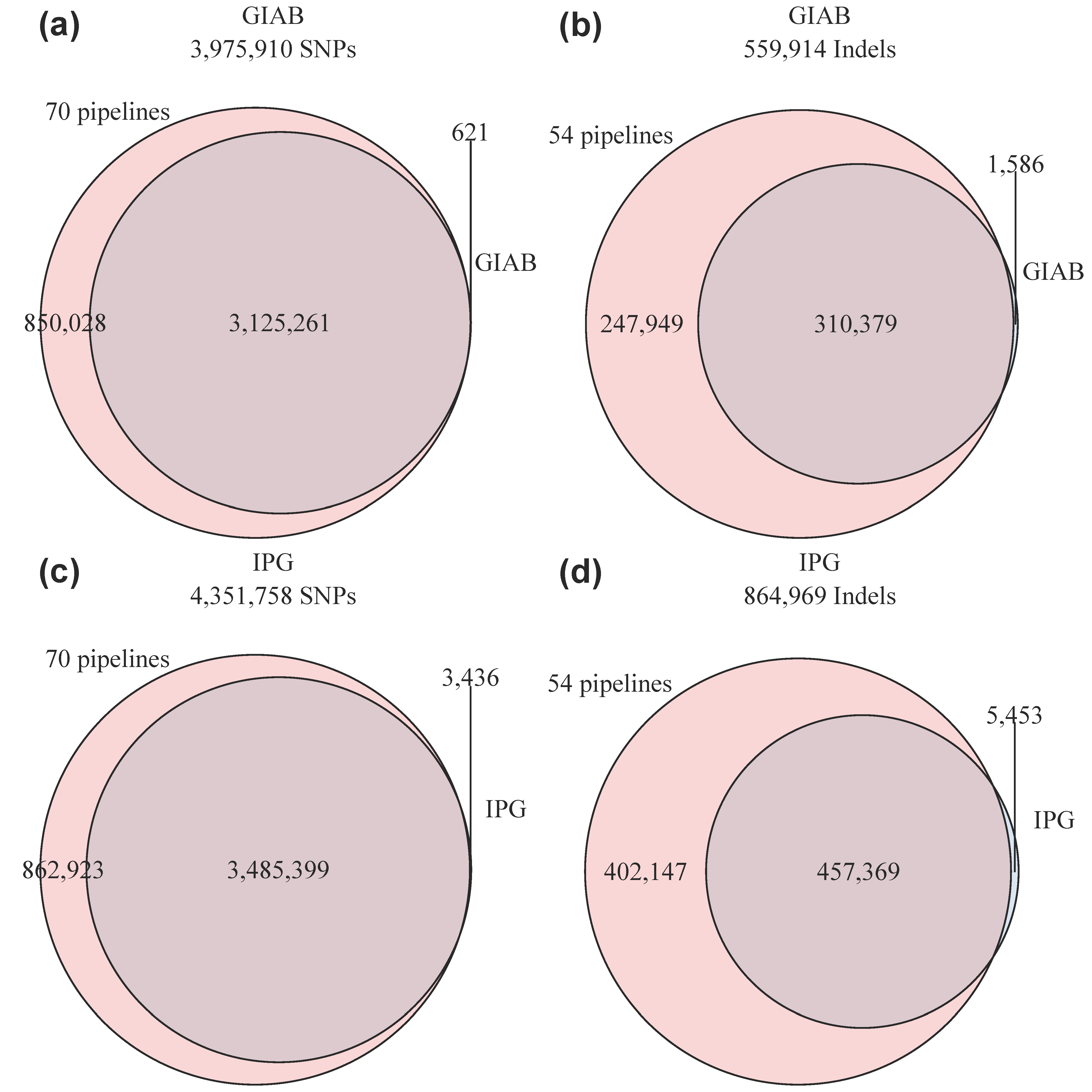


Supplementary Figure S9. Venn diagrams for comparing variant sets called by analytic pipelines and from the Genome in a Bottle (GIAB) Consortium (a and b) and the Illumina Platinum Genomes (IPG) Project (c and d).


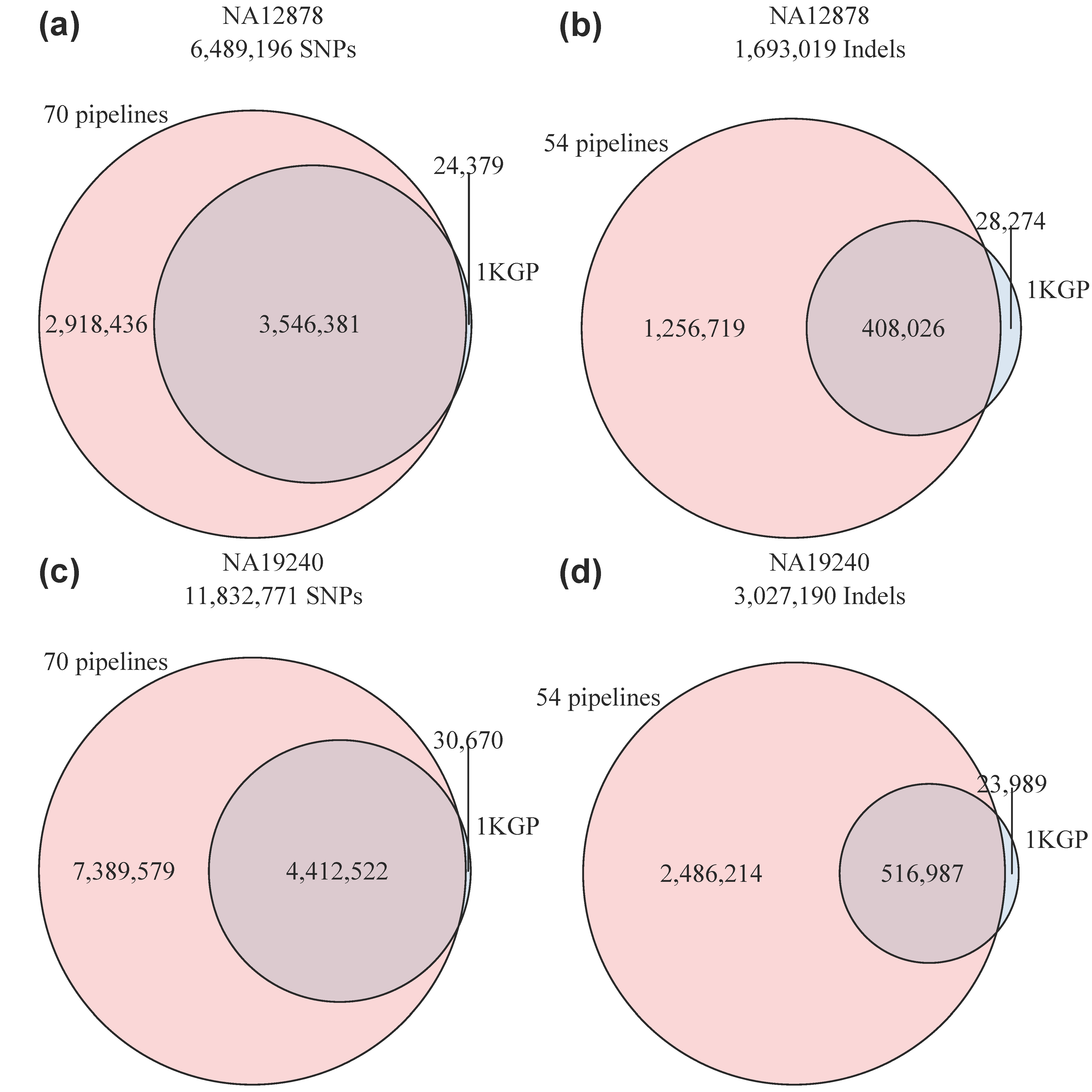


Supplementary Figure S10. Venn diagrams for comparing variant sets called by analytic pipelines and from the 1000 Genomes Project (1KGP) for NA12878 (a and b) and NA19240 (c and d).

**Logistic regression analysis to identify factors related to gold standard variant prediction**

Based on the population-specific minor allele frequencies (MAFs) from the 1000 Genomes Project (1KGP)^1^, each variant was classified as rare (MAF < 0.5%), low (0.5% ≤ MAF < 5%), common (MAF ≥ 5%), and ‘MAF not reported’. According to the severity of consequence predicted by VEP^2^, each variant was categorized into the four groups: high, moderate, low, and modifier. As sequence context, repetitive DNA elements and GC content of a variant site were used. The RepeatMasker track from the UCSC Genome Browser^3^ was used for annotating variant sites as follows: short interspersed nuclear elements (SINEs), long interspersed nuclear elements (LINEs), long terminal repeat elements, DNA repeat elements, simple repeats, low complexity repeats, satellite repeats, RNA repeats, ‘other repeats (such as rolling-circles)’, unknown, and non-repetitive elements^4^. The GC content over a window surrounding each variant site was used for annotating the site as unbiased (25–60%) or biased GC content (>60% or < 25%)^5^. The window size was set to about 2 x (‘insert size’ + 2 x ‘read length’): 1000 bps for NA12878 and 2000 bps for NA19240. Variant sites with read-depth between the first and the third quartiles for more than 80% of the short-read aligners were annotated as having normal coverage values. The average mapping quality (MAPQ) of the reads covering a variant site was calculated for each short-read aligner. Then, variant sites with an average MAPQ value larger than the median of the average MAPQ for more than 80% of the short-read aligners were annotated as having a good MAPQ value. With seven predictors, i.e., MAF and predicted functional impact of variant, repetitive DNA elements, GC bias, depth of coverage, and MAPQ at variant locus, and call concordance between analytic pipelines, logistic regression on predicting gold standard variants was performed separately for SNPs and indels, using R function glm with binomial family^6^. As gold standard variants, high confidence variants from the Genome in a Bottle (GIAB) Consortium^7^ and the Illumina Platinum Genomes (IPG) Project^8^ for NA12878, and the catalog of variants from 1KGP for NA12878 and NA19240 were used. When performing logistic regression for GIAB and IPG variants, only the variants in the respective callable regions were used.


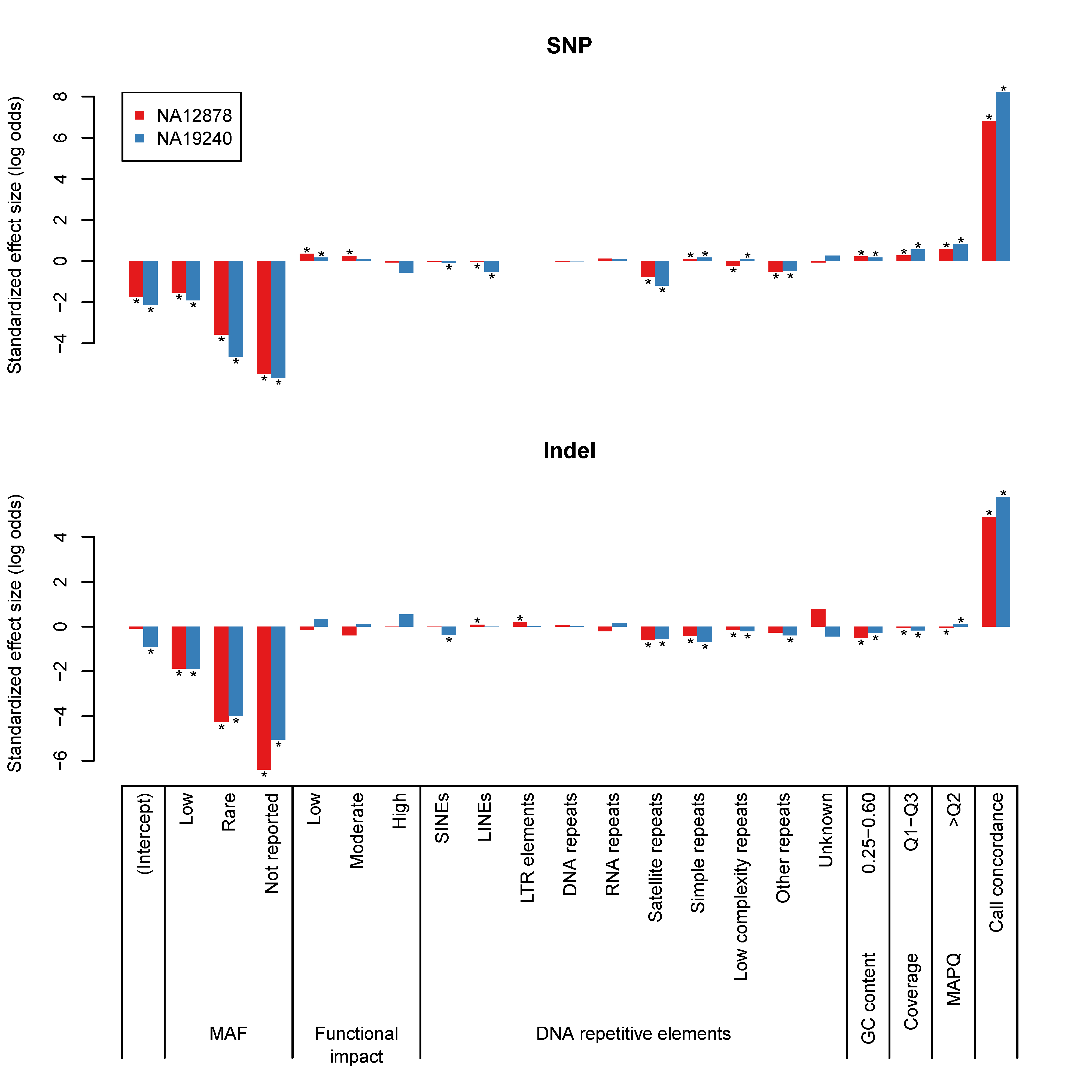


Supplementary Figure S11. Effect size of factors related to gold standard variant prediction. Variant sets from the 1000 Genomes Project for NA12878 and NA19240 were respectively used as gold standard. Logistic regression was performed using seven factors: minor allele frequency (MAF) and predicted functional impact of variants, and repetitive DNA elements, GC content, depth of coverage, and mapping quality (MAPQ) at variant loci, and call concordance between analytic pipelines. Statistically significant associations (Wald tests, *P* < 0.005) are denoted by ‘*’.


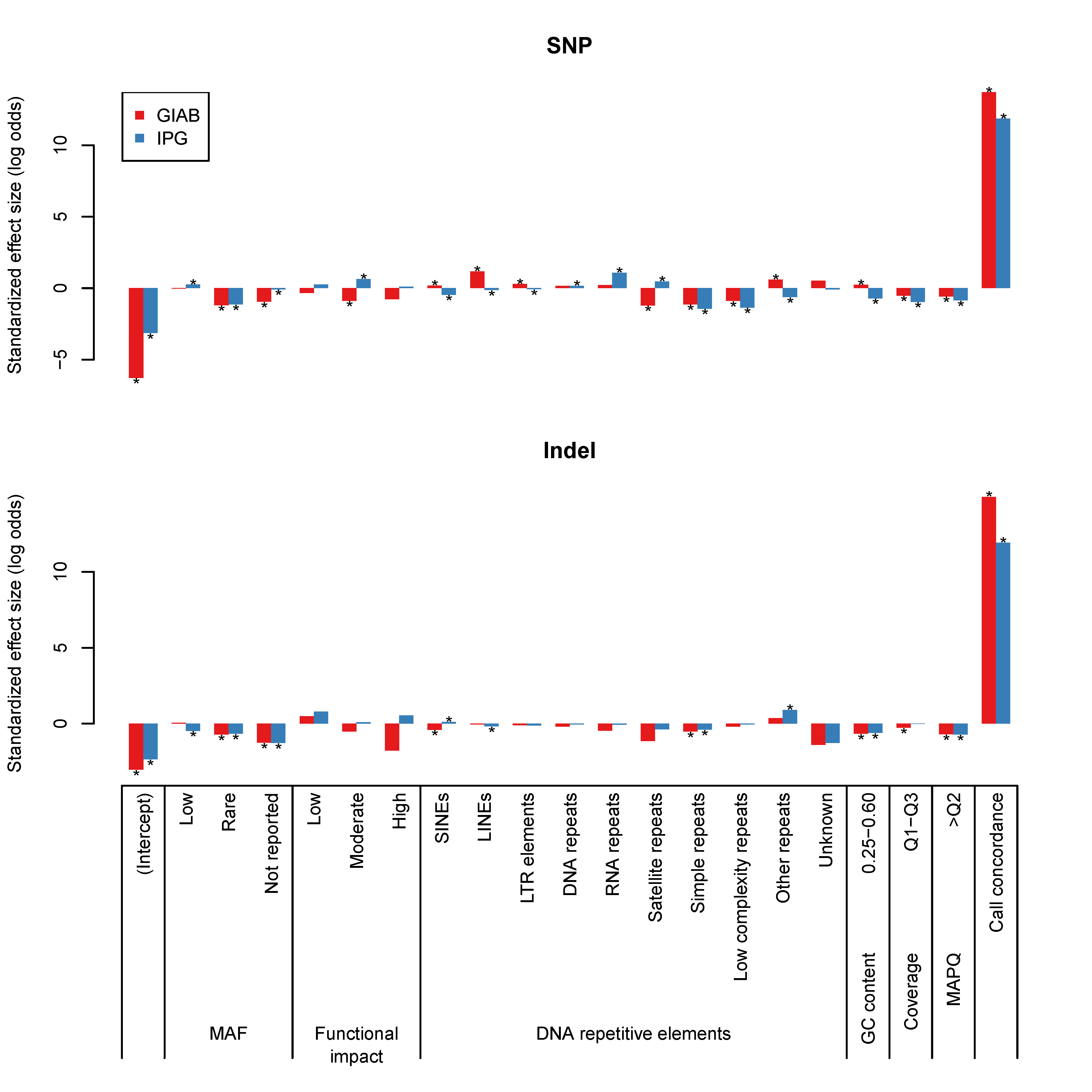


Supplementary Figure S12. Effect size of factors related to gold standard variant prediction. Variant sets from the Genome in a Bottle (GIAB) Consortium and the Illumina Platinum Genomes (IPG) Project for NA12878 were respectively used as gold standard. Logistic regression was performed using seven factors: minor allele frequency (MAF) and predicted functional impact of variants, and repetitive DNA elements, GC content, depth of coverage, and mapping quality (MAPQ) at variant loci, and call concordance between analytic pipelines. Statistically significant associations (Wald tests, *P* < 0.005) are denoted by ‘*’.

**References**

1 Genomes Project, C. *et al.* A global reference for human genetic variation. *Nature* **526**, 68-74, doi:10.1038/nature15393 (2015).

2 McLaren, W. *et al.* The Ensembl Variant Effect Predictor. *Genome biology* **17**, 122, doi:10.1186/s13059-016-0974-4 (2016).

3 Kent, W. J. *et al.* The human genome browser at UCSC. *Genome research* **12**, 996-1006, doi:10.1101/gr.229102 (2002).

4 Tarailo-Graovac, M. & Chen, N. Using RepeatMasker to identify repetitive elements in genomic sequences. *Current protocols in bioinformatics* **Chapter 4**, Unit 4 10, doi:10.1002/0471250953.bi0410s25 (2009).

5 Rieber, N. *et al.* Coverage bias and sensitivity of variant calling for four whole-genome sequencing technologies. *PloS one* **8**, e66621, doi:10.1371/journal.pone.0066621 (2013).

6 R: A Language and Environment for Statistical Computing (R Foundation for Statistical Computing, Vienna, Austria, 2017).

7 Zook, J. M. *et al.* Integrating human sequence data sets provides a resource of benchmark SNP and indel genotype calls. *Nature biotechnology* **32**, 246-251, doi:10.1038/nbt.2835 (2014).

8 Eberle, M. A. *et al.* A reference data set of 5.4 million phased human variants validated by genetic inheritance from sequencing a three-generation 17-member pedigree. *Genome research* **27**, 157-164, doi:10.1101/gr.210500.116 (2017).
